# Cure in metastatic breast cancer

**DOI:** 10.1007/s12254-018-0426-9

**Published:** 2018-08-17

**Authors:** Theresa Westphal, Simon Peter Gampenrieder, Gabriel Rinnerthaler, Richard Greil

**Affiliations:** 1 IIIrd Medical Department with Hematology and Medical Oncology, Oncologic Center, Paracelsus Medical University Salzburg, Müllner Hauptstraße 48, 5020 Salzburg, Austria; 2 Salzburg Cancer Research Institute with Laboratory of Immunological and Molecular Cancer Research and Center for Clinical Cancer and Immunology Trials, Salzburg, Austria; 3 Cancer Cluster Salzburg, Salzburg, Austria; 4 Arbeitsgemeinschaft Medikamentöse Tumortherapie (AGMT), Vienna, Austria

**Keywords:** Oligometastatic, Surgery, Chemotherapy, Targeted therapy, Immunotherapy

## Abstract

Oligometastatic disease characterizes a distinct subgroup of metastatic breast cancer patients that might benefit from different treatment strategies to achieve long-lasting remission and potentially cure. Those long-lasting remissions are reported after locoregional treatment of the primary tumor and all metastatic sites in several case series; however, unlike other tumor entities, prospective data are lacking. Furthermore, tumor eradication by excellent systemic anticancer therapy with novel chemotherapies and targeted agents can lead to long-term survival. In addition, reactivation of the host immune defense by immuno-oncologic drugs can achieve long-lasting tumor control. So far, unfortunately, checkpoint inhibitors as monotherapy have led to responses only in a small percentage of patients with metastatic breast cancer. This short review summarizes available data on long-lasting remissions and potential cure in metastatic breast cancers. It describes and discusses data on locoregional treatment, chemo-, antibody- and immunotherapy and tries to select individual patients for whom a multidisciplinary treatment approach with curative intention might be an option to achieve long-term survival.

## Introduction

Metastatic breast cancer (MBC) has been synonymous with a lethal outcome and is generally considered incurable. Although there has been an improvement in overall survival (OS) during the last decades, survival rates are still low with 5‑year and 10-year survival rates of 27% and 13%, respectively [1].

It is of great interest to define subgroups of patients suffering from this heterogeneous disease that might benefit from different treatment strategies. One special subgroup comprises patients with limited tumor spread lying between localized early breast cancer and disseminated metastatic cancer, termed oligometastatic disease. In particular, oligometastatic tumors are characterized by solitary or few metastatic lesions that are usually limited to a single organ [2]. The 3rd ESO–ESMO (European School of Oncology–European Society for Medical Oncology) International Consensus Guidelines for Advanced Breast Cancer 3 (ABC 3) extended their definition of oligometastatic disease from a solitary organ to low volume metastatic disease with limited number and size of metastatic lesions (up to five and not necessarily in the same organ), potentially amenable for local treatment, aimed at achieving long-term remission [3].

In other tumor entities the goal of cancer treatment has already moved from palliation to cure in distinct oligometastatic patient subgroups. For example in colorectal cancer, long-term remission of oligometastatic lung or liver disease is achievable in a subgroup of patients and this strategy is part of established guidelines [4].

## Definition of cure in the metastatic disease

There is no clear definition of cure in metastatic disease. The most realistic description for cure in cancer may be the following: those patients who die from other causes without any clinical evidence of cancer. As such, cure can only be defined retrospectively in an individual patient. *Cure* could also be understood as a state, where life expectancy of patients is comparable to a sex- and age-matched population. Additionally, a plateau in progression-free survival (PFS) and overall survival (OS) Kaplan–Meier curves may reflect the curative potential of a cancer treatment and serve as a surrogate for a subpopulation of patients with characteristic features.

In the commentary “International guidelines of metastatic breast cancer: can metastatic breast cancer be cured?” Pagani et al. discussed appropriate definitions and endpoints [2]. The authors state that complete remission and long-term PFS are usually surrogates for cure in metastatic cancer, but long-term survival might simply reflect an indolent nature of the disease rather than a long-term effect from therapy. Even more so, the lack of a common definition of curative treatment in the metastatic setting challenges the interpretation of the available heterogeneous data.

In order to cure metastatic disease, several approaches are imaginable: (1) locoregional treatment of the primary tumor and all metastatic sites, (2) tumor eradication by excellent systemic anticancer therapy, (3) long-term immunologic tumor control induced by immunotherapy or (4) the combination of these approaches.

## Long-term survival after surgery of distant metastases

Despite good evidence for the resection of liver metastases in other entities like colorectal cancer, data in metastatic breast cancer (MBC) are limited [5]. Several heterogeneous case series [6–20] report a wide range of survival rates between 22 and 61 months in patients with MBC undergoing liver metastases resection (Table 1). In an update of a single-center experience (*n* = 51), Ercolani et al. reported a 10-year OS rate of 16% [10]. More than half of these patients (8.9%) were alive without evidence of recurrence since surgery. However, such patient cohorts are highly selected and it is likely that not only the resection itself is responsible for the good outcome but also the indolent course of disease or the specific genetic profile of the tumor and the ability of subclones to metastasize to a certain organ. In the article “Hepatic resection for metastatic breast cancer: an exercise in selection bias” D’Ángelica concluded that retrospective case series need reasonable comparative controls in order to reduce selection bias. Prospective randomized trials would be desirable, but such data are difficult to obtain [21]. In a small prospective data collection of 41 patients undergoing resection of liver metastases, positive resection margins and a short disease-free interval (DFI) until the detection of liver metastases were identified as potential factors associated with poor long-term survival [22].

**Table 1:** Case series of resection of liver metastases in MBC published within the last 20 years

Author and reference	*n*	Survival (%)	Prognostic factors
Raab et al. 1998 [6]	34	5-year survival: 22	Negative margins (R0), no prior local recurrence
Pocard et al. 2000 [7]	52	3-year survival: 49	Long DFI
Yoshimoto et al. 2000 [8]	25	5-year survival: 27	Not reported
Pocard et al. 2001 [9]	65	4-year survival: 46	Long DFI
Elias et al. 2003 [11]	54	5-year survival: 34	Positive hormone receptor status
Vlastos et al. 2004 [14]	31	5-year survival: 61	Not reported
Sakamoto Y et al. 2005 [15]	35	5-year survival: 31	No extrahepatic disease
Adam et al. 2006 [16]	85	5-year survival: 37	Response to preoperative chemotherapy, no R2 resection, possibility of rehepatectomy in the further course of disease
Zegarac M et al. 2013 [20]	32	Median OS 37 months	Positive hormone receptor status, negative lymph nodes, long DFI, single metastases
Weinreich 2014 [12]	21	5-year survival: 33	Negative margin (R0), low primary tumor size, negative lymph nodes, low-grade histopathology, low number of liver metastases, long DFI
Ye et al. 2015 [18]	28	5-year survival: 53 10-year survival: 33	DFI >36 months, negative margins, no tumor recurrence before metastectomy
Margonis et al. 2016 [17]	131	3-year survival: 75.2	Negative margin (R0), small diameter of the liver metastasis
Kobryn et al. 2016 [19]	30	3-year survival: 36.4	Not reported
Ercolani et al. 2005 [13] and 2018 [10]	51	5-year survival: 36 10-year survival: 16	Small tumor diameter, positive progesterone receptor status, and triple negative status

*n* number of patients, *R* residual tumor, *DFI* disease-free interval, *n.g.* not given, *OS* overall survival, *MBC* metastatic breast cancer

Similar data are available for metastasectomy of pulmonary lesions [23–34]. In a systemic review including a meta-analysis, short DFI, incomplete resection of metastases, high number of metastases and negative hormone receptor status were identified as poor prognostic factors [35].

In Austria, a registry of the “Arbeitsgemeinschaft für medikamentöse Tumortherapie” (AGMT) specifically included MBC patients with surgical resection of solitary metastases and will further characterize patients with oligometastatic disease and collect survival data.

In summary, surgery of metastases is still experimental in MBC because valuable data from randomized trials and large cohorts are still missing. Even in the absence of radiologically detectable disseminated disease, MBC is in most of the cases a systemic disease and local treatment alone is not sufficient. However, the given data may help to characterize subgroups of patients with favorable outcome, where surgery could be an option in order to obtain long-term survival.

## Long-term survival induced by chemotherapy and targeted therapy

Of all patients receiving chemotherapy for MBC, only a few patients achieve a radiologic complete response (rCR) and an exceptional long remission [36, 37]. Additionally, those long-term survivors are not depicted in most MBC trials because follow-up is limited. A retrospective analysis including 75 patients aimed to characterize patients with long OS after a rCR [36]. After a median follow-up of 6 years, 28% of patients with rCR were still alive and 86% of those patients had no evidence of disease. In the multivariate analysis, anthracycline treatment and a good WHO performance status were independent predictors for long-term survival. Notably, in a historical cohort from the MD Anderson Cancer Center the percentage of long-term survivors was much lower (1–3%; [37]).

A long-term follow-up analysis reports on 285 patients with single site recurrence (76.5% locoregional, 23.5% distant) who had local treatment followed by chemotherapy. The median follow-up was 121 months and 20-year disease-free survival (DFS) and OS rates of patients with distant metastases were 18% and 21%, respectively [38]. Similar results were published by Kobayashi et al. [39]. In their retrospective analysis of multidisciplinary treatments in 75 patients with one or two organs involved (excluding the primary lesion resectable by surgery), an exceptional median OS of 185 months and a relapse-free interval of 48 months was reported. No disease progression was observed after 101 months of relapse-free survival.

Several phase III clinical trials investigating specific drugs, reported extraordinarily long median OS times. The CLEOPATRA trial, for example, reported a median OS of 56.5 months (95%CI 49.3 months to not reached) with the combination of docetaxel, trastuzumab and pertuzumab as first-line treatment in patients with human epidermal growth factor receptor 2 (HER2+) MBC [40]. In estrogen receptor (ER)+/HER2− MBC, the introduction of cyclin-dependent kinase 4/6 (CDK4/6) inhibitors led to comparable excitement. The addition to standard endocrine therapy almost doubled median PFS times in all phase III trials published today [41–46]. Currently, OS data are available from the pilot phase II PALOMA 1 trial only, where patients with ER+/HER2− MBC were treated first-line with palbociclib plus letrozole. The median OS was 37.5 months; however, some patients in this trial are still on this drug combination for now more than 6 years [47].

These trial results suggest that a few patients with MBC can achieve long-term survival (with or without complete remission of the disease), when treated with effective anticancer agents. In general, continuation of therapy is however needed to maintain this success. Therefore, effective systemic maintenance treatments with a favorable toxicity profile are needed to reach cure in the narrower sense of the word. Further long-term studies or high-level registries will help to identify and characterize those patients with exceptional outcomes induced by systemic treatment.

## Long-term survival induced by immunotherapy

The introduction of checkpoint inhibitors like anti-cytotoxic T-lymphocyte-associated protein 4 (anti-CTLA4)-, anti-programmed cell death protein 1(anti-PD1)- and anti-programmed cell death protein ligand 1 (anti-PD-L1) antibodies led to a paradigm shift in oncology. In metastatic melanoma, where overall survival was invariably short and mortality was 100%, long-term survival was reached in trials with checkpoint inhibitors. A pooled analysis of 12 trials investigating the CTLA4 antibody ipilimumab including 1861 patients with metastatic melanoma showed a 3-year survival rate of 22% (95%CI 20–24%; [48]). In trials testing anti-PD1- or anti-PD-L1 antibodies or the combination of ipilimumab and anti-PD1 antibodies, the percentage of patients with long-term survival seems to be even higher [49–51], raising the discussion about the curability of this disease. Encouraging results were also reported in non-small cell lung cancer [52–54], renal cell cancer [55] and bladder cancer [56, 57].

In MBC, only a few phase I and phase II trials investigating checkpoint inhibitors are available today ([58–62]; Table 2). Overall response rates (ORR) were moderate, but like in other diseases, durable responses in those patients responding to therapy were reported [58, 59, 63]. The longest follow-up is reported in the phase I trial Keynote-012, investigating the anti-PD1 antibody pembrolizumab as monotherapy in triple-negative MBC [58, 63]. Most of the patients were heavily pretreated with 47% of the patients having more than two prior lines of chemotherapy at study entry. The ORR was 18.5%, and the median OS was 10 months (95%CI 5.3–17.5). However, three of the five (60%) responding patients had long-lasting responses. After a median follow-up of 10.7 months, median duration of response was not reached and ranged from 15 to ≥47 weeks. One patient discontinued pembrolizumab 11 months after achieving complete remission and was still in complete remission 18 months later, without further anticancer therapy. Since the estimated response to chemotherapy in this population ranges from 4 to 12 weeks, these results are promising [64].

**Table 2:** Overall response rates (ORR) and ongoing responses in phase I–II trials with checkpoint inhibitors in metastatic breast cancer

	ER+/HER2−			TNBC		
	*n*	ORR	Ongoing responses	*n*	ORR	Ongoing responses
*Pembrolizumab (anti-PD-1)*						
Keynote-012 (phase Ib; [58])	–	–	–	32^a^	18%	11% (>1 year)
Keynote-086 (phase II) cohort A [60]	–	–	–	170	5%	0%
Keynote-086 (phase II) cohort B [59]	–	–	–	52^a^	23%	29% (>1 year)
Keynote-028 (phase Ib; [61])	25^a^	12%	0%	–	–	–
*Atezolizumab (anti-PD-L1)*						
Schmid P. (phase Ia; [62])	–	–	–	112	17%	n.g.
*Avelumab (anti-PD-L1)*						
JAVELIN (phase Ib; [65])	72	3%	4% (overall)	58	5%	4% (overall)

*ER+/HER2-* hormone receptor positive, HER2-negative*, TNBC* triple negative breast cancer, *n* number of patients, *ORR* overall response rate, *n.g.* not given

^a^Selected for PD-L1+

Checkpoint inhibitors were also tested in patients with HR-positive MBC. The phase Ib trial Keynote-028 investigating pembrolizumab monotherapy included a cohort of 25 patients with HR-positive MBC. Only 3 patients experienced partial response (ORR 12%); however, the median duration of response was 12.0 months (range, 7.4–15.9 months; [61]). In the phase Ib trial JAVELIN, 58 patients with triple-negative and 72 patients with HR-positive MBC were treated with the anti-PD-L1 antibody avelumab [65]. In the HR-positive subgroup the confirmed ORR was only 3%. Of the 47 patients with a best response of complete response, partial response or stable disease, 14 (30%) remained progression free for ≥24 weeks.

Based on results from the Keynote-012 and Keynote-086 trials, pembrolizumab will probably soon be the first checkpoint inhibitor licensed by the US Food and Drug Administration and the European Medicines Agency for the treatment of MBC. Nevertheless, because of the low percentage of patients benefiting from single-agent checkpoint inhibitor therapy, combination strategies will be required in order to increase the success rate of immunotherapy in MBC.

## Early detection of metastatic disease in an oligometastatic state

Locoregional treatment strategies are generally limited to patients with oligometastatic disease. Therefore, patients might benefit from early detection of metastatic disease after early breast cancer treatment. Using sensitive diagnostic tools like positron emission tomography/computed tomography (PET-CT; [66]) or liquid biopsies might help to detect metastasis much earlier. Liquid biopsies nowadays allow detection of circulating tumor deoxyribonucleic acid (ctDNA); [67, 68]), circulating tumor cells [69], circulating exosomes [70], circulating micro ribonucleic acids (microRNAs) [71] or tumor-educated blood platelets (TEP; [72]). However, by earlier detection, survival time of patients might falsely increase due to lead time bias [2]. Biological characteristics including molecular breast cancer subtypes, specific mutations or microRNA profiles [73] might additionally help selecting patients for certain specific therapeutic strategies leading to long-term tumor control.

## Conclusion

With improved treatment options and individualized treatment strategies *cure* might be an achievable goal for highly selected patients with MBC. Specifically, in oligometastatic disease, where a combination of local and systemic treatment is feasible, such long-term effects can be achieved. According to the current literature, long-term disease-free survival can be observed after surgery of distant metastases; however, the biology of the disease has to be considered when patients are selected for such an approach. Unfortunately, valuable comparative data are still missing, for which reason surgery of breast cancer metastases still remains experimental and an individual decision. Chemotherapy, as well as targeted therapies, can lead to long-lasting disease control. Since it is unknown if systemic therapy can be stopped in case of long-lasting complete remission, therapy is generally continued. Therefore, a favorable toxicity profile is one of the requirements for a systemic therapy with curative potential. Reactivation of the host immune defense by immuno-oncologic drugs can achieve long-lasting tumor controls. Unfortunately, checkpoint-inhibitors as monotherapy lead to responses only in a small percentage of patients with MBC. Therefore, combination strategies are needed in order to increase the probability for tumor shrinkage, long-term responses and finally cure.

Since for all of these strategies low volume disease increases the success rate, early detection of metastatic disease might be one step forward. New technologies in imaging and liquid biopsies could help in this regard. Finally, only selection of patients for individualized treatment options in an interdisciplinary environment will help to establish a cure for MBC in the future.
